# Potential Use of Mobile Phone Applications for Self-Monitoring and Increasing Daily Fruit and Vegetable Consumption: A Systematized Review

**DOI:** 10.3390/nu11030686

**Published:** 2019-03-22

**Authors:** Floriana Mandracchia, Elisabet Llauradó, Lucia Tarro, Josep Maria del Bas, Rosa Maria Valls, Anna Pedret, Petia Radeva, Lluís Arola, Rosa Solà, Noemi Boqué

**Affiliations:** 1 Functional Nutrition, Oxidation, and Cardiovascular Diseases Group (NFOC-Salut), Facultat de Medicina i Ciències de la Salut, Universitat Rovira i Virgili, 43201 Reus, Spain; floriana.mandracchia1@urv.cat (F.M.); elisabet.llaurado@urv.cat (E.L.); rosamaria.valls@urv.cat (R.M.V.); anna.pedret@urv.cat (A.P.); 2 Unitat de Nutrició i Salut, Centre Tecnològic de Catalunya, Eurecat, 43204 Reus, Spain; josep.delbas@eurecat.org (J.M.d.B.); lluis.arola@urv.cat (L.A.); noemi.boque@eurecat.org (N.B.); 3 Facultat de Matemàtiques i Informàtica, Universitat de Barcelona, 08007 Barcelona, Spain; petia.ivanova@ub.edu; 4 Centre de Visió per Computador, 08193 Bellaterra (Barcelona), Spain; 5 Nutrigenomics Research Group, Department of Biochemistry and Biotechnology, Universitat Rovira i Virgili, 43007 Tarragona, Spain; 6 Hospital Universitari Sant Joan de Reus, 43204 Reus, Spain

**Keywords:** mobile app, mHealth, fruits, vegetables, self-monitoring, healthy diet

## Abstract

A wide range of chronic diseases could be prevented through healthy lifestyle choices, such as consuming five portions of fruits and vegetables daily, although the majority of the adult population does not meet this recommendation. The use of mobile phone applications for health purposes has greatly increased; these applications guide users in real time through various phases of behavioural change. This review aimed to assess the potential of self-monitoring mobile phone health (mHealth) applications to increase fruit and vegetable intake. PubMed and Web of Science were used to conduct this systematized review, and the inclusion criteria were: randomized controlled trials evaluating mobile phone applications focused on increasing fruit and/or vegetable intake as a primary or secondary outcome performed from 2008 to 2018. Eight studies were included in the final assessment. The interventions described in six of these studies were effective in increasing fruit and/or vegetable intake. Targeting stratified populations and using long-lasting interventions were identified as key aspects that could influence the effectiveness of these interventions. In conclusion, evidence shows the effectiveness of mHealth application interventions to increase fruit and vegetable consumption. Further research is needed to design effective interventions and to determine their efficacy over the long term.

## 1. Introduction

The health benefits of consuming fruits and vegetables have been extensively demonstrated. These beneficial effects are attributed to their high contents of fibre, vitamins, minerals and phytochemicals (mainly antioxidants) together with negligible amounts of fat. Increased consumption of fruits and vegetables has been associated with reduced risks of many chronic diseases, such as obesity, cardiovascular diseases, type II diabetes, osteoporosis and certain cancers, as well as all-cause mortality [1,2]. In fact, it was estimated that in 2013, 7.8 million premature deaths worldwide could be attributed to low fruit and vegetable intake [1]. Moreover, adequate intake of fruits and vegetables could avoid approximately 31% of ischaemic heart disease, 19% of stroke, 20% of oesophageal cancer and 19% of gastric cancer cases [3].

Health authorities, such as the World Health Organization (WHO), recommend a daily intake of at least 400 g of fruits and vegetables, which corresponds to 5 servings of 80 g per day [4]. For the purpose of encouraging fruit and vegetable consumption in all women, children and men so they meet the recommended intake, the WHO launched an international programme termed “5 a day”, which has been adopted by most national governments, including Spain, France, Germany and the United Kingdom. Similarly, the Dietary Guidelines for Americans advise that one-half of the plate should be fruits and vegetables [5], and Canada’s Food Guide recommends including plenty of vegetables and fruits in daily meals and snacks to prevent the risk of heart diseases [6].

International organizations and national governments have set increasing fruit and vegetable intake as a priority. Despite the numerous and diverse public health campaigns implemented in recent decades to promote increased consumption of fruits and vegetables in Western countries, the average intake remains far from these recommendations, reflecting the modest impact of these kinds of interventions. Data from a European Food Safety Authority (EFSA) analysis based on national dietary surveys revealed that only 4 of the European Union (EU) member states reported adequate consumption of fruits and vegetables [7]. The success of the last major campaigns conducted worldwide that intended to increase fruit and vegetable consumption has been reviewed by Rekhy et al. [8], who concluded that these interventions were quite effective in the short term but generally failed over the long term despite the enormous cost and effort they require. Importantly, it is inferred from the same work that the effectiveness of these health programmes is greater when factors such as behavioural changes, goal setting, clear messages and interactive approaches are included.

In recent years, strategies for promoting long-term adherence to different interventions have focused on multidisciplinary approaches. For example, management of weight loss depends on multiple factors, such as behaviour, a cognitive component, personality traits and even the patient-therapist interaction [9]. This multifaceted approach has been proven successful for weight loss maintenance over the long term (up to 42 months) by means of coaching strategies [10]. Integrative health coaching conducted by telephone calls has been used as a tool for enhancing treatment outcomes in type 2 diabetic patients, who are able to improve their adherence to medication and glycated haemoglobin, a marker of long-term blood glucose levels [11]. Johnson et al. showed that health coaching delivered by videoconference was an effective strategy for reducing weight and ameliorating insulin-resistance markers in obese individuals [12]. Overall, health coaching and behavioural changes have arisen as key elements for achieving substantial and long-term adherence to healthy habits. Therefore, such approaches represent a promising strategy for increasing the consumption of fruits and vegetables.

In this scenario, current advances in information and communication technologies (ICTs), also known as eHealth [13], might provide a wide array of supportive tools, allowing a wide deployment of coaching and behavioural change strategies to the general population. Importantly, it is inferred from the same work that the effectiveness of these health programmes is greater when factors such as self-monitoring, goal setting, clear messages and interactive approaches are included. MHealth applications, which are used on mobile phones and wireless devices, such as tablets, personal digital assistance (PDA) devices, and so on, could be a better method to improve people’s lifestyles [13] than traditional face-to-face education methods [14]. The mHealth App Developer Economics study showed an increase of 25% year-to-year from 2015 to 2017 of the number of mHealth applications [15]. Moreover, from the mHealth Economics report, an increase from 2.1 billion smartphone users in 2016 to 2.5 billion in 2019 [16] is expected. Mobile technologies allow interactions with users in real-time and the delivery of health interventions at any time [17] and can act in different environmental and behavioural contexts [17]. Mobile technologies have been demonstrated to be a valid tool for dietary self-monitoring [18]. Toro-Ramos et al. showed that using a mobile phone application that provides nutritional and behavioural education together with coaching promoted clinically significant long-term weight loss, reduced blood glucose levels and improved different lipid markers in overweight and obese individuals [19]. There are several basic mobile and web journaling applications that allow users to set weight-loss goals, collect daily calorie target chart data to reflect trends over time, and record food consumption and exercise levels. The indicative paradigms of journaling applications are weight management applications, such as Weightbot© (2017 Meeco Labs, Linz, Austria), LoseIt© (2008–2019 FitNow, Inc, Saint Honoré, Paris), InsideTracker© (2009–2019 Segterra, Inc, Cambridge, MA, U.S.A.), FoodLog© (2013 foo.log, Inc, Tokyo, Japan), Cronometer© (2011–2019, Cronometer.com, Revelstoke, BC, Canada), MyFitnessPal© (2009–2019 MyFitnessPal, Inc, San Francisco, CA, U.S.A.), MyPlate© (2017 LIVESTRONG.COM, Santa Monica, CA, U.S.A.), EasyFit© (2016 Cellularline, Reggio Emilia, Italy), FatSecret© (2019, FatSecret, Victoria, Australia), MyNetDiary© (2018 MyNetDiary Inc, Marlton, NJ, U.S.A.), and so on, which enable the user to enter weight and body composition measurements, visualize curves, superimpose trends and track progress. Following standard paper-based analogues to obtain information on nutrition habits, mobile applications provide electronic forms and efficient interfaces to assist in logging food intake and beverages in terms of types, meal courses, total meal calories, recipes, photos, and so on. These applications calculate energy intake and balance, report additional parameters and visualize summaries [20]. Three systematic reviews demonstrated the efficacy of mHealth applications to prevent obesity in young people [21,22,23], but there is a lack of scientific evidence of the effects on fruit and vegetable consumption, while the majority of published studies focus on weight management and physical activity improvement [24]. Thus, new technologies represent a promising opportunity in fields such as nutrition and health monitoring [25,26,27].

Increasing and improving the consumption of fruits and vegetables in the general population represents a challenge for public health that has not yet been resolved. ICTs might represent an opportunity to achieve this objective. Therefore, we conducted a systematized review of the last 10 years to assess whether interventions based on mobile phone applications result in positive outcomes and to identify the main weaknesses of the different approaches used to date.

## 2. Materials and Methods

The present paper is a systematized review and has some characteristics of a narrative review and some of a systematic review [28].

### 2.1. Search Strategy

Article searches were limited to a recent time range of 10 years, considering that the use and availbility of mobile phone applications, which are the key tools evaluated in the present review, increased only a few years ago, starting from 2007, when they appeared on the market. In this sense, there is a lack of published trials on the use of mobile phone applications for health interventions before 2010 [29,30,31]. This systematized review was based on two electronic databases: PubMed and Web of Science. The search strategy involved peer-reviewed and English-language articles. For the search strategy, the following keywords were used separately or in combination: ‘Self-monitoring’ AND ‘Fruit and Vegetables’ OR ‘Healthy meals’, ‘Fruit and vegetables’ AND ‘Mobile health applications’ OR ‘eHealth’ OR ‘mHealth’ OR ‘Mobile technology’, and ‘Mobile phone applications’ AND ‘Fruit and Vegetables’.

### 2.2. Selection Criteria and Data Collection

**Figure 1:**
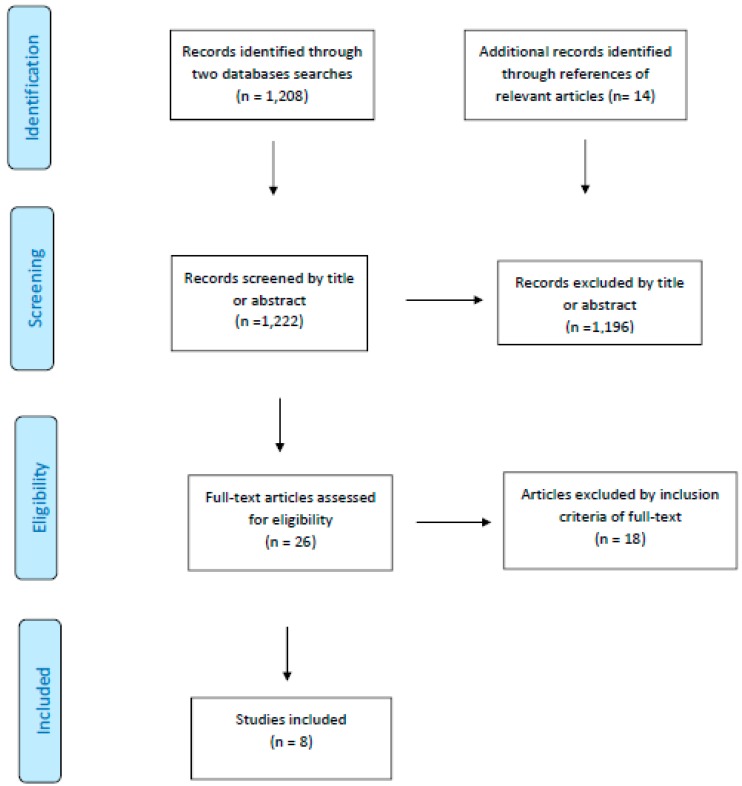
Preferred Reporting Items for Systematic Reviews and Meta-Analyses (PRISMA) 2009 flow diagram for the systematic review and meta-analysis of the article selection process.

The PubMed and Web of Science databases were searched, resulting in a total of 1208 articles, as shown in the PRISMA (Preferred Reporting Items for Systematic Reviews and Meta-Analyses) flow diagram for systematic reviews and meta-analysis (Figure 1); of these articles, 228 were found in MEDLINE (PubMed) and 980 in Web of Science. During the screening of possible articles, the reference lists of the full-text articles were assessed for eligibility and cross-checked; it was decided to evaluate articles from these lists when they focused on the same outcome, resulting in the identification of 14 articles for further screening. The titles and abstracts of the 1222 total articles were screened by two researchers (F.M. and E.L.) to determine if they fulfilled the eligibility criteria. The articles had to include clinical trials and other experimental studies designed to develop, test or validate a mobile phone application for dietary self-monitoring in which fruit and/or vegetable intake was one of the principal outcomes (primary and/or secondary). Studies including other health concerns (physical activity, weight control, sugar-sweetened beverage intake, takeout meals, dietary habits, etc.) were included, and no limitations were made in terms of the type of population (gender, age, race, health status). Studies using web-based self-monitoring technologies were excluded, which focused the search on mobile phone applications only. This selection process was performed by two reviewers (F.M. and E.L.). In cases of discrepancy, a third reviewer (L.T.) was consulted.

Following the screening, 1196 articles were excluded on the basis of their title or abstract. The remaining 26 articles were subjected to a detailed examination of the abstract to determine their eligibility on the basis of the inclusion criteria. Of these, 18 were excluded due to the type of technology tool used or lack of results, leaving 8 peer-reviewed papers included in the current review.

### 2.3. Data Extraction

Data extraction from the included studies was performed by two reviewers working simultaneously (F.M. and E.L.) and revised by all others. The data extraction tables include the following study variables: name of the article, authors, year of publication, name of the intervention or mobile phone application, country, type of intervention, objective, intervention duration, number of participants, population, description of the mobile phone application (name and measurement of the outcome), brief description of the intervention, results of fruit and vegetable consumption variables, and conclusions of the study.

### 2.4. Quality of Studies Included

The quality of the included studies was assessed using the standardized framework of the Quality Assessment Tool for Quantitative Studies, developed by the Effective Public Health Practice Project. This tool consists of 8 items: selection bias, study design, confounders, blinding, data collection methods, withdrawals and dropouts, intervention integrity and analysis. This tool allows the categorization of each study’s methodological quality as weak (≥2 weak category ratings), moderate (1–3 strong category ratings and 1 weak category rating) or strong (≥4 strong category ratings and no weak category ratings) (Table S1).

## 3. Results

The systematized search identified 1222 articles, of which eight studies were found to meet the inclusion criteria to be considered in the review (Table S2).

The most relevant characteristics of the data extracted from the included studies are presented in Table 1 and Table S2: Relevant information regarding the data extracted from the included studies.

The eight studies included were randomized controlled trials (RCTs) [32,33,34,35,36,37,38,39], one of which was a pilot study [32], and included a total of 1524 participants at baseline. Additionally, the participants covered a large age range, from 16 to 71 years, and one of the interventions was conducted through parents, although the target population was children. In the vast majority of cases, the participants did not present any disease. Most of the studies were conducted in the United States (*n* = 4), followed by Australia (*n* = 2), the Netherlands (*n* = 1) and Sweden (*n* = 1).

From the selected studies, two studies aimed to evaluate the effectiveness of mobile phone applications in stimulating both fruit and vegetable intake [34,38], two targeted only vegetable consumption [32,33], three tested whether a multicomponent intervention integrating a mHealth application could improve dietary habits (including the increase of fruit and/or vegetable intake) and physical activity [35,37,39], and only one assessed the effectiveness of a mobile phone application to achieve a healthy weight and healthy body fat percentage by changing daily servings of fruits and vegetables [36].

Moreover, different methodologies were used to improve fruit and/or vegetable intake: (a) three of the studies used personalized informative and motivational messages (text and/or audio) [34,38,39]; (b) seven added personal dietary feedback at a regular frequency [32,33,34,35,36,37,38]; (c) three sent push notifications to remind users about their goals [32,33,36]; (d) two provided rewards as incentives [35,37]; (e) three provided remote coaching support through mobile phone calls, emails and in-person meetings [35,37,39]; (f) one offered the possibility of receiving support from a dietitian or a psychologist [36]; and (g) two provided access to further informative material and information through a diet booklet [39] and a mobile phone application [36,39].

Fruit and/or vegetable intake was assessed by the Food Frequency Questionnaire (FFQ) in three RCTs [32,33,34], by a dietary record in one study [35], by self-monitoring through a mobile phone application in two studies [36,38], and by categorical questions in the other two studies [37,39]. Moreover, the results were expressed as servings, pieces/day or pieces/week of fruits and/or vegetables in six RCTs [32,33,34,35,37,38]; grams of fruits and/or vegetables in one RCT [36]; and percentage of participants who consumed ≥2 servings or pieces of fruits and/or vegetables per day in one RCT [39].

Six of the eight studies included were effective in increasing fruit and/or vegetable intake. Of these, five studies [32,33,35,37,39] demonstrated that the interventions were effective for increasing vegetable consumption, and interestingly, all of them included a self-monitoring component implemented by a mobile phone application. Some of the included studies used other methodologies apart from self-monitoring: four used dietary feedback [32,33,35,37] and three provided remote coaching support [35,37,39]. Furthermore, three studies [34,35,37] reported that the interventions were effective for increasing daily fruit consumption. All three mHealth interventions included a self-monitoring component by mobile phone application and personal dietary feedback, while two of them [35,37] provided a financial incentive as a reward and remote coaching support. The increase in intake ranged from +2.4 servings/day to +10.6 servings/day.

Moreover, from the effective interventions identified in the present review, two focused on overweight adults [32,33], three focused on adults with unhealthy lifestyles [34,35,37], and one focused on young adults characterized by unhealthy lifestyles [39].

Half of the included studies considered the improvement of fruit and/or vegetable intake [32,33,34,38] as the primary outcome, and the other half considered it to be a secondary outcome [35,36,37,39].

Of the four RCTs that designated increasing fruit and/or vegetable intake as the primary outcome [32,33,34,38], two were only effective in increasing vegetable intake [32,33], one showed was only effective in increasing fruit intake [34], and the last one presented no improvement [38]. These four RCTs lasted from 2 to 6 months and were population stratified by common characteristics.

Mummah et al. have iteratively developed a theory-driven mobile phone application called Vegethon to increase vegetable consumption through self-monitoring, goal setting, feedback, and social comparison [40]. Vegethon has been tested in two studies: A RCT pilot study [32] and a RCT [33]. The target population of the Vegethon pilot study comprised 17 overweight adults aged 18–50 years [31,32] who were randomized for the use of the Vegethon mobile phone application as the intervention group or to a wait-listed control condition. The intervention group was instructed to use the Vegethon application and encouraged to self-monitor and increase their vegetable intake. At 12 weeks, the results showed that vegetable intake was significantly increased in the intervention group by +7.5 servings/day (from 6.0 ± 2.7 to 13.5 ± 8.1) compared with the decrease in the control group of −3.1 servings/day (from 7.0 ± 5.9 to 3.9 ± 2.0), resulting in a significant difference of +10.6 servings/day between both groups (*p* = 0.02). Moreover, as mentioned, the effectiveness of Vegethon in increasing vegetable consumption was also verified in an RCT among 135 overweight adults aged 18–50 years [20]. The intervention was the same as in the pilot study. The intervention group reported an increase of +0.7 servings/day of vegetables (from 6.7 ± 5.2 to 7.4 ± 5.4), while the control group reported decreased vegetable consumption of −1.7 servings/day (from 8.1 ± 8.2 to 6.4 ± 4.3), resulting in a significant difference of +2.4 servings/day between both groups (*p* = 0.04). As a result, the Vegethon mobile phone application was effective in improving vegetable consumption.

### 3.1. Increasing Daily Fruit and/or Vegetable Consumption as the Primary Outcome

The study by Elbert et al. [34] provides evidence-based insight into the effects of a mobile health application in changing fruit and vegetable intake in a 6-month intervention. This study was a 3-arm RCT that included a population of 146 adults aged 16–71 years. The intervention groups (A and B) were exposed monthly to tailored health information and feedback in the form of either (A) an audio-based intervention or (B) a text-based intervention via mobile phone application over a 6-month period. Participants in the control group only completed the baseline and post-intervention measures. After 6 months, the average fruit intake, measured by a food frequency questionnaire, increased by +3.3 pieces/week (from 14.2 ± 10.6 to 17.5 ± 11.1) in intervention group A, whereas in intervention group B, the average fruit intake decreased by −0.6 pieces/week (from 14.8 ± 11.1 to 14.2 ± 6.9), and in the control group, the average fruit intake increased by +0.4 pieces/week (from 13.4 ± 10.4 to 13.8 ± 9.4). However, the intake of vegetables was not improved by these interventions.

In another 3-arm RCT, the Connecting Health and Technology study [38], Kerr et al. aimed to evaluate the effectiveness of tailored dietary feedback and weekly text messaging to improve the dietary intake of fruits and vegetables among other dietary improvements over a 6-month period in a population-based sample of men and women aged 18–30 years. Participants were randomized into three groups: (A) a group that received dietary feedback and weekly text messages, (B) a group that received dietary feedback only and (C) a control group (that received any intervention). Dietary intake was assessed using a mobile food record application in which participants captured images of the foods and beverages they consumed over 4 days at baseline and at 6 months post-intervention. After 6 months of intervention, participants in group B and the control group demonstrated a significantly increased daily intake of vegetable servings (+0.4 ± 0.1, *p* = 0.002 and +0.4 ± 0.1, *p* = 0.02, respectively), while group A demonstrated a significantly decreased daily intake of fruit servings (−0.2 ± 0.1; *p* = 0.03). However, no significant differences between groups in terms of fruit and vegetable intake were observed (*p* < 0.05).

### 3.2. Increasing Daily Fruit and/or Vegetable Consumption as a Secondary Outcome

Of the four RCTs that designated increasing fruit and/or vegetable intake as the secondary outcome [35,36,37,39], one revealed that the intervention was effective in both targets [37], two were partially effective [35,39], and the last one was not effective for either of the two targets [36]. These four RCTs lasted from 3 to 9 months, and all of the RCTs were population stratified.

The Make Better Choices (MBC) study [35] was a comparative 4-arm RCT designed to discern the optimal approach to simultaneously target diet and physical activity. The MBC study [35] consisted of a 6-month intervention (3-week intervention and 5-month follow-up) with 204 adults aged 21–60 years who were randomized into one of four behavioural change prescriptions. The MBC study compared four different behaviours: (1) 5 fruit/vegetable servings; (2) saturated fat consumption of less than 8% of total calories; (3) physical activity of at least 60 min/day; and (4) sedentary leisure of less than 90 min/day. The intervention consisted of present and remote coaches accessed by a mobile personal digital assistant (PDA) that tailored the behavioural strategies based on the baseline data of the participants. Moreover, participants received financial incentives when they reached the goals. The two groups targeted to increase fruit and vegetable intake (Group B and Group C) seemed more successful than the other two groups targeted to change other behaviours: Group B increased from 1.3 ± 1.1 servings/day at baseline to 5.6 ± 1.1 servings/day at the end of the intervention, and Group C increased from 1.2 ± 0.9 servings/day at baseline to 5.5 ± 1.0 servings/day at the end of the intervention. The two groups that were not targeted to improve fruit and vegetable intake (Group A and Group D) seemed less successful than the other two groups: Group A increased from 1.1 ± 0.9 servings/day at baseline to 1.7 ± 1.1 servings/day at the end of intervention; Group D increased from 1.4 ± 1.1 servings/day at baseline to 1.9 ± 1.6 at the end of intervention. However, the differences between baseline and the end of intervention regarding fruit and vegetable intake in each group and among groups were not reported by the researchers.

Another study related to the MBC study was the MBC 2 trial [37], a 3-arm RCT that tested whether a multicomponent intervention of 9 months (6-month intervention and 3-month follow-up) integrating a mHealth application, modest incentives and remote coaching could sustainably improve dietary habits and physical activity. Participants were randomly assigned to one of two interventions. Intervention group (A) targeted performing moderate to vigorous physical activity (MVPA) simultaneously with other diet and activity targets, and intervention group (B) targeted the same goals but sequentially. The control intervention group only addressed improving stress and sleep. After 6 months, fruit and vegetable intake increased by +6.6 servings/day in group A (simultaneous), by +7.4 servings/day in group B (sequential) and by +0.5 servings/day in the control group.

In the third study, Partridge et al. [39] performed a 2-arm RCT from a larger mHealth lifestyle program called “TXT2BFiT” to improve dietary and physical activity behaviours among 248 young adults aged 18–35 years who were at high risk for the development of obesity. The intervention group comprised 8 weekly motivational text messages, 5 personalized coaching calls, 1 weekly email, a diet booklet and a mobile phone application that provided education, self-monitoring, access to a community blog and support resource, over 3 months. Control group participants only received 4 text messages and dietary and physical activity guidelines. Intervention participants were more likely to consume greater quantities of vegetables after 3 months compared to control participants (*p* = 0.009). Additionally, at 3 months, the proportion of participants with a vegetable intake of ≥2 servings/day increased from 34.1% to 64.3% in the intervention group and from 36% to 48% in the control group.

The Mobile-Based Intervention to Stop Obesity in Pre-schoolers (MINISTOP) [36] aimed to help, through intervention by their parents, 315 children aged 4.5 years to improve their body status, nutritional habits and physical activity via a smartphone application during a 6-month intervention. Participants were randomly assigned to the intervention or control group: the intervention group received a 6-month mHealth application to register information about their child’s food consumption and physical activity; and the control group received a pamphlet on healthy eating and physical activity. The differences between baseline and the follow-up for the intervention group resulted in an increase of 2.9 ± 78.9 g/day of fruits and −6.7 ± 42.1 g/day of vegetables consumed, while for the control group, decreases of −12.1 ± 87.9 g/day of fruits and −3.6 ± 39.7 g/day of vegetables were observed. However, no significant differences between groups were observed in fruit or vegetable intake.

### 3.3. Quality Appraisal and Risk of Bias in the Included Studies

Analysis of the quality of the included studies showed that all of the studies were of weak quality (≥2 weak category ratings). The best good quality items were the study design and dropouts, whereas the other items were of poor quality.

## 4. Discussion

The present review aimed to investigate the current literature on the potential use of mobile phone applications to self-monitor and increase the intake of fruits and/or vegetables. From a search of the literature, eight studies were included in the final screening for evaluation. The present review proposes that mobile phone applications that include a self-monitoring component have great potential in improving fruit and vegetable intake, supporting the important health benefits associated with technology-based interventions. Six of the eight studies included in the review were effective. These studies focused on overweight adults and adults or young adults with unhealthy lifestyles. Thus, it could be inferred from the current review that the interventions delivered through mobile phone applications that successfully improved the intake of fruits and/or vegetables had something in common: stratification of a specific population that has common interests and motivations. Focusing on the age of the participants, it was observed that 5 studies targeting the adult population (18–60 years) were effective, 2 studies targeting young adults (18–35 years) were not effective or partially effective, and 1 study targeting children through their parents was not effective. Age seems to be an irrelevant factor in determining the effectiveness of the intervention, while other factors, such as participants’ common interests, play a key role in achieving an increase in fruit and vegetable intake. Indeed, population selection is one of the characteristics considered in social marketing principles to enable healthy choices [41]. Moreover, two [32,33] of the six effective studies focused on increasing fruit and/or vegetable intake in targeted overweight adults, suggesting that the effectiveness of these types of interventions could be influenced by specific motivations, such as overweight-associated health risks. Pre-existing health problems in the study population were related to increased effectiveness of the intervention compared with the effectiveness observed in the population without diseases, as demonstrated in a previous review [23].

Furthermore, it seems that both outcomes, increased fruit and vegetable daily intake, were better achieved when self-monitoring and dietary feedback were used in the intervention. However, two of the analysed RCTs that failed to increase fruit and vegetable consumption [36,38] also included these methodologies together with push notifications or motivational text messages. These contradictory results could be explained because in one of these studies, parents were responsible for improving the fruit and vegetable consumption of their children [36]; thus, it seems that monitoring the dietary intake of children via their parents is not effective in improving dietary habits.

On the other hand, increasing fruit and/or vegetable intake was not the primary outcome for all of the eight included studies. Four studies had decreasing body weight or body fat or improving diet and activity behaviours as their primary outcome, while increasing fruit and/or vegetable intake was set as a secondary outcome [35,36,37,39]. In these trials, the effects on fruit and/or vegetable intake evaluated as a secondary outcome were unclear. Thus, our results suggested that when the increase of fruit and/or vegetable intake was defined as the primary outcome, the intervention was more effective than when it was defined as a secondary outcome.

All of the eight studies included in this review implemented a self-monitoring and self-reporting component through a mobile phone application to set and control users’ daily fruit and/or vegetable intake goals. Considering the other parts of the methodologies of the studies included in the review, all the studies were randomized controlled studies ranging from two to nine months of intervention and reported the need for further investigations to observe the effects over time. From the results presented in this systematized review, it was observed that an increase of fruit and/or vegetable intake could be observed from two to nine months, and an early rise in vegetable intake compared to that in fruit intake was found, which required more time to achieve an effective improvement.

The increased amount of fruit and/or vegetable intake is an important point to be discussed. Considering that one serving is equal to a minimum of 80 g [42], the minimum increase achieved in the eight studies included in the present review of +2.4 servings/day is an approximately 200 g increase in fruit and/or vegetable intake. Accordingly, an increase of 200 g/day of fruit and/or vegetable intake is associated with an 8%–16% reduction in the relative risk of coronary heart disease, 13%–18% reduction in the risk of stroke, 8%–13% reduction in the risk of cardiovascular diseases, 3%–4% reduction in the risk of cancer, and 10%–15% reduction in the risk of all-cause mortality [1].

The use of mHealth applications has increased, but the question of whether these applications are better than traditional methods is still open. Users have demonstrated general acceptability and adherence to mobile phone tools [43,44] in comparison with the traditional methods of dietary self-monitoring [45,46] because of their personal tailoring, low cost, and interactivity [47]. Furthermore, self-monitoring though mobile phone applications seems to provide easier and real-time dietary assessments [48] and is also associated with better quality dietary data compared with traditional methods, which could be affected by users’ memory [44]. However, more evidence is needed for mHealth applications because the majority of these applications are developed with minimum feedback users and little support [49]. Although self-monitoring via mobile phone applications seems to have positive effects on fruit and/or vegetable intake, its relationship with an effective improvement of dietary habits has not yet been confirmed. Although mobile phone applications have been widely tested in weight-loss trials [24,50], their utilization for the improvement of specific target food group intake is still scarce.

Comparing different methodologies used to increase the consumption of fruits and/or vegetables, a systematic review from 2005 that considered 44 studies using diverse approaches, but not mobile phone applications, observed increases in fruit and vegetable intake from 0.1 to 1.4 servings/day in heathy adults [23]. Computer-tailored information and interventions using telephone contacts were found to represent an adequate alternative to face-to-face education and counselling-based interventions [23]. Notably, the improvements in fruit and/or vegetable intake observed in the studies included in the present review (range: +0.2 to +7.5 servings/day of fruit and vegetable), which used mobile phone applications, seem to be greater than those obtained from interventions employing more traditional methodologies.

Finally, although the use of different mobile phone applications in several studies [9,11,19,51] shows a positive outcome in increasing the awareness of the quality of food intake, improving dietary habits and educating individuals, it is clear that the implementation of mHealth applications for fruit and/or vegetable intake promotion can deeply affect the final outcome. Moreover, it seems that only monitoring fruit and/or vegetable intake may not be sufficiently engaging; thus, implementing smart techniques for individual engagement, such as expert feedback [9,10,12,19] or positive rewards, could affect the final success of the mobile phone application [12,19]. In other words, it is well known that the effectiveness of mHealth applications depends on the usability their interface, feedback, rewards, and so on. A complete comparison would require using different mobile phone applications and studying their usability and effectiveness in the same population group. Unfortunately, most of the mobile phone applications used are built in-house and thus are not publicly available for direct comparison. Moreover, implementation of an extensive usability study is beyond the scope of this paper.

Additionally, assessment of food intake through web and mobile app tools requires the collaboration of individuals and thus can be subjective, retrospectively biased, and suffer from low compliance. It is tedious for people to continuously annotate their food intake over long periods of time. In addition to the fact that having to annotate all meals is embarrassing and subjective, people generally do not remember all the food they have eaten. Another important drawback of manual annotation is food underreporting [20]. Moreover, many health applications are not created by nutritional professionals. Additionally, we cannot assume that food diaries based on personal annotations of a few days are representative of an individual’s complete diet [20]. Recently, some web-based and mobile applications have included automatic food recognition that is based on smartphone pictures. Some applications have very recently claimed to introduce this option: LoseIt!© (2008–2019 FitNow, Inc., Saint Honoré, Paris), MyFitnessPal© (2009–2019 MyFitnessPal, Inc., San Francisco, CA, U.S.A.), CalorieMama© (2017, Azumio, Inc., Redwood city, CA, U.S.A) and FatSecret© (2019, FatSecret, Victoria, Australia). This ability makes the process of food intake reporting easier, faster and more pleasant, but it currently suffers from not being able to recognize a large amount of foods in the diet, demonstrating sub-optimal performance and limited recognition of different types of dishes.

In general, the majority of the articles included in the present systematized review discussed future interventions, such as larger-scale and longer trials, rather than technical improvements of mHealth applications. Regardless, some design elements could be taken into account for the future development of mHealth applications to improve vegetable and fruit intake, such as (a) inclusion of a validated tool to register food intake and to improve dietary assessments [38]; (b) weekly messages to reinforce the health recommendations about vegetable and fruit intake [39]; (c) remote connected coaching [37]; (d) remainders to buy fruits and vegetables when people are in the supermarket (e) interactive information between users and coaches [34,35]; and (f) tracking and sensor technologies as an interactive information system [34].

There are several limitations in this review. First, the majority of the trials included in this review presented the following limitations: the population sample was not representative of the community setting because of the small size, level of education, gender and origin [32,33,34,35,36,38,39] and low reliance of the data, which were self-reported by participants [32,33,35,36,37,38,39]. Second, the reviewed studies expressed results on the primary outcome (fruit and/or vegetable intake) using different units of measure, such as g/day, servings/day, pieces/week and percentage of people consuming ≥2 servings/day. Third, a description of the amount of grams considered to be a serving of fruit and/or vegetable intake was not provided. Fourth, the self-monitoring tool type was not the same for all the studies, which influenced the presentation of the final results (servings/day or week, g/day, percentage, etc.), and could be better expressed in future studies as servings/day or g/day. Fifth, only two databases were used to search for results: PubMed and Web of Science. Although these databases are the most commonly used, the inclusion of other databases could have increased and influenced the final results of the review. Sixth, the present paper is a systematized review, i.e., the review process is shorter than that of a systematic review, may or may not include comprehensive searching, may or may not include quality assessment, and describes the uncertainty around the findings and the limitations of the methodology [28]. Finally, although the study quality was not an inclusion criterion, the weakness of the majority of the included studies presents problems for the generalizability of the results of this systematized review.

## 5. Conclusions

The present review demonstrates that effective interventions to increase fruit and vegetable consumption using mobile phone applications last from two to nine months and are characterized by a stratified population that shares the same motivation to achieve better dietary habits. Furthermore, the inclusion of behavioural change techniques, such as dietary feedback together with self-monitoring and remote coaching support, has been identified as a key element that can definitively facilitate the adoption of new dietary habits. This issue strongly suggests that behavioural theory-based strategies must be considered when designing dietary mHealth application interventions. Further research on mHealth applications is needed to design more effective interventions and to determine their efficacy over the long term. Although evidence shows a promising future for mHealth applications to promote healthy nutrition, it is an open question as to how to ensure that the maturity and popularity of these applications is similar to those of other tools for the promotion of healthy habits, such as activity trackers.

## Figures and Tables

**Table 1:** Description of the studies included in the present review.

Reference	Study Design	Duration	Type of Intervention and Target Population	Outcome	Effectiveness	Change in Fruit Consumption	Change in Vegetable Consumption
[32]	Randomized Controlled Pilot Study	3 months	Self-monitored by mobile-phone application. Directed to overweight adults aged 18–50. Total: 17 participants (intervention: *n* = 8; control: *n* = 9).	Primary	**✓**For vegetables	Fruit consumption not measured	**Intervention group:** +7.5 servings/day; **Control group:** −3.1 servings/day; **Difference between groups:** 10.6 servings/day
[33]	Randomized Controlled Trial	2 months	Self-monitored by mobile-phone application. Directed to overweight adults aged 18–50. Total: 135 participants (intervention: *n* = 68; control: *n* = 67).	Primary	**✓**For vegetables	Fruit consumption not measured	**Intervention group:** +0.7 servings/day; **Control group:** −1.7 servings/day; **Difference between groups:** 2.4 servings/day
[34]	Randomized Controlled Trial	6 months	Self-monitored by mobile-phone application, text-based or audio-based health messages. Directed to adults aged 16–71 who had not yet succeeded in consuming the daily recommended fruit and vegetable consumption. Total: 146 participants (intervention: *n* = 88; control: *n* = 58).	Primary	**✓**For fruits **x** For vegetables	**Textual feedback condition:** −0.6 pieces/ week; **Auditory feedback condition:** +3.3 pieces/week; **Control group:** +0.4 pieces/week; **Differences between Textual- Control:** −0.2; **Differences between Auditory-Control:** 3.7	Non-significant difference
[35]	Randomized Controlled Trial (4-arm parallel groups)	6 months (3 weeks of treatment and 20 weeks of follow-up)	Self-monitored by a PDA, remote coach support and financial incentives. Directed to adults aged 21-60 who eat <5 servings of fruits and vegetables a day, consume ≥8% daily calories from saturated fat; do <150 min/week of Physical Activity and watch >120 min/week of leisure screen time. Total: 204 participants (group A: *n* = 47; group B: *n* = 48; group C: *n* = 56; group D: *n* = 53).	Secondary	**✓**For fruits and vegetables (only significant improvements in Behaviour C intervention)	**Baseline vs. 3 weeks** **Behaviour A (targeted saturated fat and physical activity):** +0.6 servings/day; **Behaviour B (targeted fruit/vegetable intake and physical activity):** +4.3 servings/day; **Behaviour C (targeted fruit/vegetable intake and sedentary behaviours):** +4.3 servings/day; **Behaviour D (targeted saturated fat and sedentary behaviour):** +0.5 servings/day	
[36]	Randomized Controlled Trial	6 months	Self-monitored, informed and supported by a mobile-phone application, weekly graphic feedback and facultative dietitian contact for further support. Directed to parents of children aged 4.5. Total: 315 participants (intervention: *n* = 156; control: *n* = 159).	Secondary	**x** For fruits **x** For vegetables	**Intervention group:** +2.9 ± 78.9 g/day; **Control group:** −12.1 ± 87.9 g/day	**Intervention group:** −6.7 ± 42.1 g/day; **Control group:** −3.6 ± 39.7 g/day
[37]	Randomized Controlled Trial (3-arm parallel groups)	9 months (6 months of treatment and 3 months of post-intervention follow-up)	Self-monitored by a mobile-phone application and an accelerometer, remote coaching by telephone and rewarding financial incentives. Directed to adults aged 18–65 characterized by those who eat <5 servings of fruits and vegetables a day, consume ≥8% daily calories from saturated fat; do <150 min/week of Physical Activity and watch >120 min/week of leisure screen time. Total: 212 participants (intervention A: *n* = 84; intervention B: *n* = 84; control: *n* = 44).	Secondary	**✓**For fruits and vegetables	**Baseline vs. 6 months** **Group A (Simultaneous):** +6.6 servings/day; **Group B (Sequential):** +7.4 servings/day; **Control group:** +0.5 servings/day.	
[38]	Randomized Controlled Trial (3-arm parallel groups)	6 months	Self-monitored by a mobile-phone application, dietary feedback messages and text messages via mobile-phone. Directed to young adults aged 18–30. Total: 212 participants (intervention A: *n* = 84; intervention B: *n* = 84; control: *n* = 44).	Primary	**x** For fruits **x** For vegetables	**Group A:** −0.2 ± 0.1 servings/day; **Group B:** −0.1 ± 0.1 servings/day; **Control Group:** −0.2 ± 0.1 servings/day; **Between group difference in Mean change (*p* > 0.05)**	**Group A:** +0.2 ± 0.1 servings/day; **Group B:** +0.4 ± 0.1 servings/day **Control Group:** +0.4 ± 0.1 servings/day; **Between group difference in Mean change (*p* > 0.05)**
[39]	Randomized Controlled Trial	3 months	Self-monitored and trained by a mobile-phone application, text messages, e-mails, coach calls, diet booklet and access to resources. Directed to young adults aged 18–35 characterized by fruit intake <2 servings/day and vegetable intake <5 servings/day. Total: 248 participants (intervention: *n* = 123; control: *n* = 125).	Secondary	**✓**For vegetables **x** For fruits	Non-significant difference	Intervention group: from 34.1% (baseline) to 64.3% (12 weeks) of people consuming ≥2 servings/day; **Control group:** from 36% (baseline) to 48% (12 weeks) of people consuming ≥2 servings/day

****✓**** Effective in increasing fruit or vegetable consumption (mentioned in the table). **x** No effective in increasing fruit or vegetable consumption (mentioned in the table).

## Supplementary Materials

The following are available online at https://www.mdpi.com/2072-6643/11/3/686/s1, Table S1: Quality of the studies included in the systematized review; Table S2: Relevant information regarding the data extracted from the included studies.
