# Supplementary material for: Potential Use of Mobile Phone Applications for Self-Monitoring and Increasing Daily Fruit and Vegetable Consumption: A Systematized Review
Source: Nutrients. 2019 Mar 22;11(3):686. doi: 10.3390/nu11030686 (PMC6471011; doi:10.3390/nu11030686)
Supplement: Supplementary file 1 [file nutrients-11-00686-s001.zip › nutrients-435986-Table S1.docx]

**Table S1. Quality of articles included in the systematized review**

| **AUTHOR, YEAR AND TITLE** | **SELECTION BIAS** | **STUDY DESIGN** | **CONFOUNDERS** | **BLINDING** | **DATA COLLECTION METHODS** | **WITHDRAWALS AND DROPOUTS** | **QUALITY** |
| --- | --- | --- | --- | --- | --- | --- | --- |
| Mobile Technology for Vegetable Consumption: A Randomized Controlled Pilot Study in Overweight Adults | 3 | 1 | 2 | 2 | 3 | 2 | WEAK |
| Effect of a mobile app intervention on vegetable consumption in overweight adults: a randomized controlled trial | 3 | 1 | 2 | 2 | 3 | 1 | WEAK |
| A Mobile Phone App Intervention Targeting Fruit and Vegetable Consumption:The Efficacy of Textual and Auditory Tailored Health Information Tested in a Randomized Controlled Trial | 2 | 1 | 2 | 2 | 2 | 3 | WEAK |
| Multiple Behavior Changes in Diet and Activity  A Randomized Controlled Trial Using Mobile Technology | 3 | 1 | 1 | 2 | 2 | 1 | WEAK |
| Mobile-based intervention intended to stop obesity in preschool-aged children: the MINISTOP randomized controlled trial | 3 | 1 | 2 | 2 | 3 | 2 | WEAK |
| Multicomponent mHealth Intervention for Large, Sustained Change in Multiple Diet and Activity Risk Behaviors: The Make Better Choices 2 Randomized Controlled Trial | 2 | 1 | 2 | 2 | 3 | 1 | WEAK |
| The connecting health and technology study: a 6-month randomized controlled trial to improve nutrition behaviours using a mobile food record and text messaging support in young adults | 2 | 1 | 2 | 3 | 3 | 1 | WEAK |
| Effectiveness of a mHealth Lifestyle Program With Telephone Support (TXT2BFiT) to Prevent Unhealthy Weight Gain in Young Adults: Randomized Controlled Trial | 3 | 1 | 1 | 2 | 3 | 1 | WEAK |

*Cochrane Handbook for Systematic Reviews of Interventions using the standardized framework of the Quality Assessment Tool for Quantitative Studies, developed by the Effective Public Health Practice Project.*
